# Use of non-growing *Lactococcus lactis* cell suspensions for production of volatile metabolites with direct relevance for flavour formation during dairy fermentations

**DOI:** 10.1186/s12934-014-0176-2

**Published:** 2014-12-10

**Authors:** Bert van de Bunt, Peter A Bron, Lolke Sijtsma, Willem M de Vos, Jeroen Hugenholtz

**Affiliations:** TI Food and Nutrition, Wageningen, The Netherlands; NIZO food research, Ede, The Netherlands; The Kluyver Centre for Genomics of Industrial Fermentations/NCSB, Delft, The Netherlands; Wageningen UR Food & Biobased Research, Wageningen, The Netherlands; Laboratory of Microbiology, Wageningen University, Wageningen, The Netherlands; Department Veterinary Biosciences, University of Helsinki, Helsinki, Finland; University of Amsterdam, Swammerdam Institute of Life Sciences, Science park 904, PO Box 94216, 1090 GE Amsterdam, The Netherlands

**Keywords:** Flavour, Lactococcus lactis, Lactic acid bacteria, Non-growing, Amino acid metabolism, Screening method, Cheese

## Abstract

**Background:**

*Lactococcus lactis* is a lactic acid bacterium that has been used for centuries in the production of a variety of cheeses, as these bacteria rapidly acidify milk and greatly contribute to the flavour of the fermentation end-products. After a short growth phase during cheese ripening *L. lactis* enters an extended non-growing state whilst still strongly contributing to amino acid-derived flavour formation. Here, a research approach is presented that allows investigation of strain- and amino acid-specific flavour formation during the non-growing state.

**Results:**

Non-growing cells of five selected *L. lactis* strains were demonstrated to degrade amino acids into flavour compounds that are relevant in food fermentations and differs greatly from production of flavour compounds using growing cells. As observed earlier in other research set-ups and with other microorganisms, addition of NADH, α-ketoglutarate and pyridoxal-5-phosphate was demonstrated to be essential for optimal flavour formation, suggesting that intracellular pools of these substrates are too low for the significant production of the flavour compounds. Production of flavours during the non-growing phase strongly depends on the individual amino acids that were supplied, on the presence of other amino acids (mixtures versus single compounds), and on the strain used. Moreover, we observed that the plasmid-free model strains *L. lactis* MG1363 and IL1403 produce relatively low amounts of flavour components under the various conditions tested.

**Conclusions:**

By using this simplified and rapid approach to study flavour formation by non-growing lactic acid bacteria, lengthy ripening periods are no longer required to assess the capacity of strains to produce flavours in the long, non-growing state of dairy fermentation. In addition, this method also provides insight into the conversion of single amino acids versus the conversion of a mixture of amino acids as produced during protein degradation. The generated results are complementary to earlier generated datasets using growing cells, allowing assessment of the full flavour forming potential of strains used as starter cultures in industrial food fermentation processes.

## Background

Fermented dairy products contain a wide range of flavours resulting from biochemical conversions of milk ingredients such as caseins, milk fat and lactose. Particularly in cheese, the lactic acid bacterium *Lactococcus lactis* contributes significantly to the degradation of casein and many cheese flavours are formed as a result of these proteolytic processes and, more specifically, the conversion of amino acids [1-3]. *L. lactis* can import small peptides derived from this extracellular proteolysis. Intracellular peptidases convert these small peptides into amino acids which are subsequently converted to flavour compounds [4-7]. Many of the observed differences in the flavour of various cheese types are related to the presence of different (combinations of) *L. lactis* strains [8-11]. During cheese ripening, cells reach a non-growing state after a couple of days while they continue to produce flavour compounds [12,13]. The time needed and the process towards the non-growing state are strain-specific and have been shown to affect the flavour formation [14]. During cheese ripening, a multitude of flavours are formed via various conversion routes and the process of flavour formation is, generally, slow. Therefore, monitoring this process is quite labour-intensive. Although it is clear that specific *L. lactis* strains possess different flavour-producing activities [15], the exact biochemical explanation for the differences is not fully understood. This is mainly due to the fact that many of the relevant microbial pathways have not yet been characterized or have not been studied under the relevant, non-growing conditions found in cheese. Due to the long time periods required for cheese ripening it is not feasible to distinguish which observed conversions are purely chemical or are directly related to the presence of microbial enzymes. In addition, in actual cheese the formation of compounds can only be followed cumulatively from beginning to the end of the ripening period and direct extraction of active microorganisms or microbial enzymes from cheese has proven to be quite difficult if not impossible [16,17]. For this reason, various approaches, often with different objectives, have been designed to study cheese flavour development. These include measuring flavour-profiles of cheeses in time by using Gas Chromatography in combination with Mass Spectrometry (GCMS) [18-20]. In specific cases, this method is preceded by steam distillation [21,22]. However, sampling during ripening is tedious and time-consuming. The so called CH-easy model was developed where concentrated cells of lactic acid bacteria were added to a young industrial cheese, in which the endogenous bacteria have been inactivated [23]. This system has been applied in various studies that allowed simultaneous analysis of multiple strains of lactic acid bacteria. Measurements in cheese and cheese-paste give insight into the complete mix of flavours that can be produced by certain lactic acid bacteria but it will not allow the investigation of specific enzyme and substrate reactions, in relation to specific flavour production. For this reason many specific enzyme activities obtained from lactic acid bacteria growing in cheese were studied, such as proteinase and peptidase activities [24-28]. In addition, individual enzymes involved in amino acid catabolism such as transferases, decarboxylases and dehydrogenases have been intensively studied [24,29-32]. These enzyme activities can be measured in cell free extracts [29,33], whereas in the case of the cell wall proteinase, enzyme activity can be measured in whole cells [25,34]. The combination of nuclear magnetic resonance and GC is an elegant way to study amino acid catabolism [29] but is limited to the analysis of labelled compounds. Flavour formation by cheese starter bacteria could also be studied during the first 24 h of a milk fermentation process, until the cells reach the stationary phase of growth, but this approach will exclude all flavour formation in the subsequent non-growing phase, which is the physiological state of the starter bacteria during most of the ripening period. Mass spectrometry metabolic fingerprinting is reported to be a comprehensive and sensitive approach to study flavour formation in a model cheese made with ultra-filtrated milk concentrate [35]. For all these models it can be argued that the experimental set-up does not represent actual cheese conditions and that the models do not allow direct *in situ* analysis or that the models involve growing cells, whereas the major part of the cheese ripening process involves non-growing cells. Moreover, the studies with the real cheese-like models, such as the CH-easy model, various curd models or cheese juices, are still hampered by difficulty/impossibility of perturbations and extractions. Hence, there is a clear need for alternative, improved, approaches.

Earlier studies by the group of Mireille Yvon and others [36-40] have shown that resting, non-growing, cells of various microorganisms actually convert amino acids into flavour components. The conversion of the branched chain amino acid L-Leucine to isovaleric acid, for example, was studied in a phosphate buffer using resting cells and cell free extracts of *Propionibacterium*. In this same study it was shown that addition of α-ketoglutarate was essential for the conversion of L-Leucine to α-ketoisocaproic acid by *P.freudenreichii* [39]. Using a similar experimental set-up, the ability of resting, non-starter, Lactobacilli and thermophilic lactic acid bacteria to produce various flavour compounds from amino acids were studied [40]. In these studies it was also shown that addition of α-ketoglutarate is not required for flavour formation by (resting cells of) *Streptococcus thermophilus* and that flavour formation in actual Cheddar [34] and semihard cheese [35] is stimulated by addition of α-ketoglutarate.

In this manuscript, we describe a research approach, based on the work of Yvon and co-workers, to study the role of, non-growing, *Lactococcus lactis* in flavour development during cheese ripening. The approach allows acceleration of the flavour-forming process by using concentrated cell suspensions and various perturbations and extractions of samples for analysis without major impact on cell physiology. In this study, we did not include the role of the lysis of starter bacteria, although we are fully aware of the importance of lysis in actual cheese ripening [17]. Since the method that we describe here requires only hours of incubation versus weeks and months during cheese ripening, we can assume that lysis will play a much smaller role during buffer incubations than in actual cheese manufacturing. We focussed on some aspects that were not previously studied, such as the role of the (growth) history of the *L. lactis* cells on the ability to produce flavour components, the strain variability in flavour formation and the difference between simultaneous conversion of various amino acids versus conversion of single amino acids. Furthermore, we demonstrated that biological variance in these flavour-generating experiments can be significantly reduced using frozen cell suspensions deriving from one large fermentation instead of producing new cell material for every individual experiment.

Overall, the analyses reveal important strain- and amino acid availability-dependent flavour formation by *Lactococcus lactis*.

## Results and discussion

### Experimental setup

An experimental set-up was used to determine the flavour-forming potential of various *L. lactis* strains under non-growing conditions. Ten-fold concentrated cell suspensions were made by suspending the cell pastes of the various strains with a final OD600 of 40. By using these 10 fold concentrated cell suspensions relatively rapid detection of flavour formation became possible. Our cell incubation system was validated using *L. lactis* strain 1157, well-known for its production of 3-methylbutanal and 3-methylbutanol. This strain was incubated in sodium phosphate buffer containing leucine and NADH for 24 h at 30°C followed by GCMS analysis of the volatile metabolites (Table 1). It was observed that mere incubation of cells in phosphate buffer with various amino acids was not sufficient for the production of 3-methylbutanal or 3-methylbutanol. We observed some formation of these volatiles in the incubation mix with only leucine and glucose (no cells), probably as a result of chemical conversion, i.e. Strecker reaction. In the incubations with cells and sugar (0.2%), we observed significant growth of the bacteria and volatile production which is not related to the production in the non-growing state. Hence, to ensure that the cells were not growing during incubation, sugar addition was aborted for all remaining experiments. In the presence of α-ketoglutarate, we found a nine fold increase in the level of the sum of the aldehyde and alcohol. Extra addition of pyridoxal-5-phosphate led to a tenfold increase of these same flavour compounds as compared to the incubation of just leucine and cells. Rapid freezing (in liquid nitrogen) storage at −40°C and thawing in a water bath at 30°C had no particular effect on the production of volatiles when the concentrated cells (optical density of 400 at 600 nm) were incubated in 100 mM sodium phosphate buffer (pH 6.0) with 15% glycerol (data not shown). This allowed the usage of a constant cell biomass which is essential for performing technical replicates. The finding that addition of α-ketoglutarate, and pyridoxal-5-phosphate was required to achieve a high flavour production is consistent with findings of previous studies where α-ketoglutarate was required to confirm aminotransferase activity [32] and enhancement of flavour compound production in Cheddar cheese [36]. Hence, we have used these additions in all remaining experiments.

**Table 1:** **Development of incubation mixture for volatile production by non-growing cells**

	**3-methylbutanal**	**Fold change**	**3-methylbutanol**	**Fold change**
leucine + cells	1.0E + 08	1.0	2.2E + 07	1.0
leucine + glucose	6.4E + 06	0.1	3.8E + 06	0.2
leucine + glucose + cells	2.5E + 08	2.4	5.7E + 07	2.6
leucine + α-ketoglutarate + cells	3.6E + 08	3.6	7.7E + 08	35.2
leucine + α-ketoglutarate + pyrydoxal-5-phosphate + cells	6.2E + 08	6.2	5.9E + 08	27.1

Fold change is displayed against the incubation with leucine and cells.

Effect of α-ketoglutarate and pyridoxal phosphate on 3-methyl butanal and 3-methyl butanol production (GCMS-peak area) by *L.lactis* 1157 in a buffer system containing 100 mM phosphate, pH 6,0, 2 mM NADH and 10 mM leucine.

### Strain dependent flavour profile of non-growing strains

**Figure 1:**
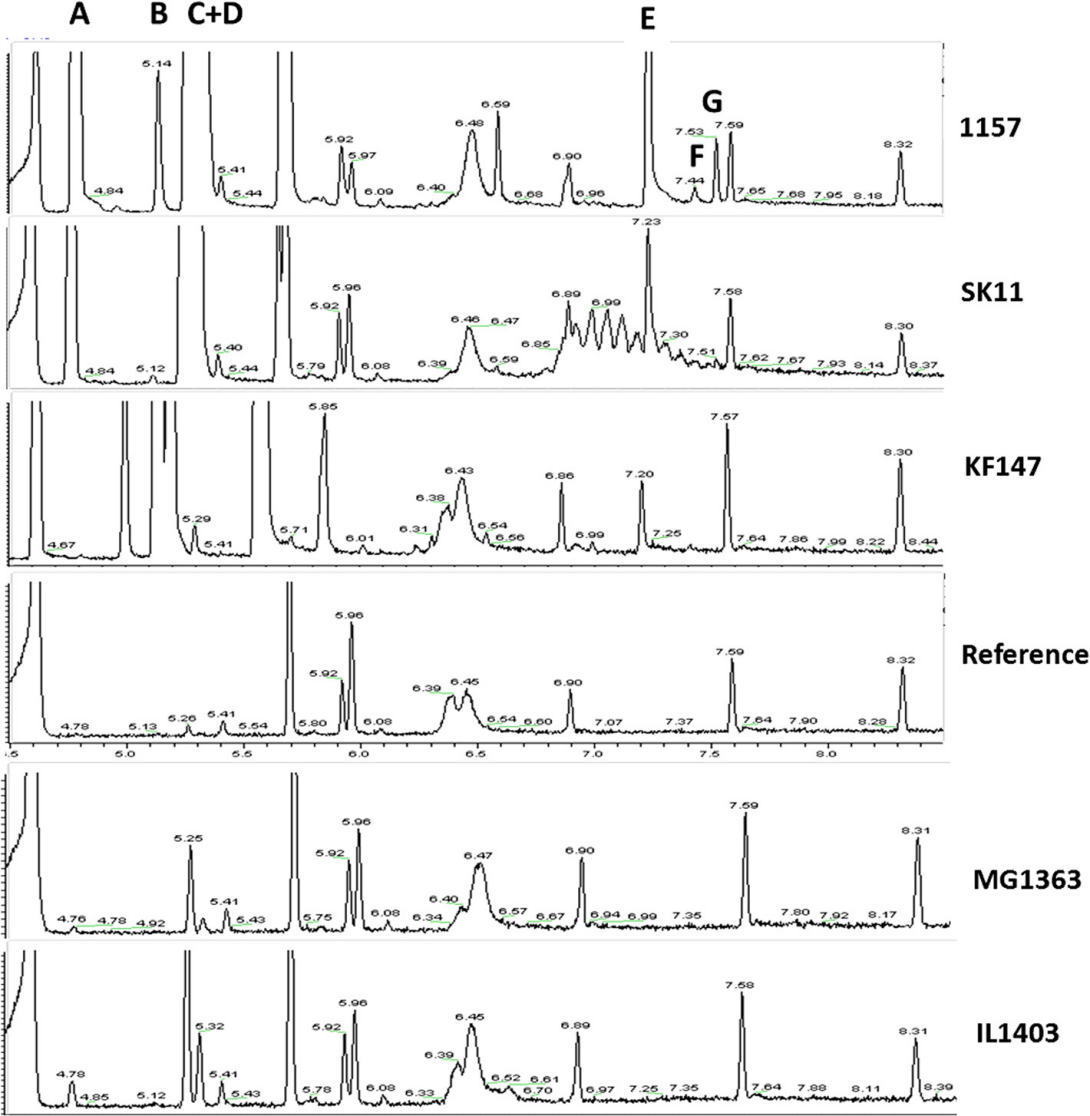
**Volatile compound production of non-growing** ***Lactococcus lactis*** **strains.** *L.lactis* KF147, SK11, 1157, MG1363 and IL1403 in a buffer system containing 100 mM sodium phosphate, pH 6.0, 2 mM NADH, 10 mM α-ketoglutarate, 0.1 mM pyridoxal phosphate and the amino acid mixture. Displayed volatile compounds: **A**: 2-methylpropanal, **B**: 2-methylpropanol, **C**: 3-methylbutanal, **D**: 2-methylbutanal, **E**: phenylacetaldehyde, **F**:3-methylbutylvalerate, **G**: 2-isopropyl-5-methyl-2hexenal.

Subsequently, the buffer incubation system was used to compare volatile production by five different *L. lactis* strains. Clear differences were observed in production of some important volatiles as highlighted in the GCMS profiles (Figure 1). All strains produced a range of volatile components, although there were obvious differences in production levels of volatiles and in variety of volatiles produced.

All strains produced volatile compounds, although two strains, *L. lactis* MG1363 and IL1403 were only observed to produce 2-methylpropanal, 3-methylbutanal, and 2-methyl butanal, all products from branched chain amino acid conversions [41]. Strains SK11, 1157, and KF147 exclusively produced the volatile 2-methylpropanol, likely to be a direct result of valine conversion. These three strains also produced phenylacetaldehyde from phenylalanine degradation whereas MG1363 and IL1403 did not produce this flavour compound, indicating that phenylacetaldehyde production is a result of microbiological activity and not of chemical conversion in these incubation mixtures. There were also clear differences observed in flavour profiles when SK11, 1157, and KF147 were compared. For example in the conversion of leucine and isoleucine, strain 1157 produced the highest amounts of the flavours 3-methylbutanal and 2-methylbutanal. Moreover, it was observed that strain SK11 produced the aldehyde 2-methylpropanal but not the corresponding alcohol 2-methylpropanol, whereas this aldehyde and alcohol were found in incubations with strain 1157 and KF147. Strain 1157 uniquely produced two fruity flavours: 2-isopropyl-5-methyl-2-hexenal and 3-methylbutylvalerate.

These data on strain-specific amino acid conversions reiterate the importance of strain selection for industrial fermentations where good and distinct flavours are the main objective.

### Influence of growth phase and amino acid availability on flavour formation

To assess the influence of the bacterial growth phase on flavour formation, cells of *L. lactis* strain 1157 were studied at two different time-points during cultivation, in the growing and the non-growing phase in a buffer system (Table 2). It was observed that 2-butanone, acetic acid, acetone, diacetyl and DMDS were only produced by growing cells. In contrast, 2-methylpropanol, methylbenzoate and some of the esters such as *n*-butyl isovalerate, 3-methylbutyl-2-methylbutyrate, 2-isopropyl-5-methyl-2-hexanal and 4-methyl-2-phenyl-2-hexenal were only produced by non-growing cells. Benzaldehyde, phenylacetaldehyde [42] methylbenzoate, and 5-methyl-2-phenyl-2-hexenal, all products of aromatic amino acid conversions were clearly produced at higher levels by non-growing cells compared to growing cells. However, 2-methylpropanal, 2-methylbutanal, 3-methylbutanal, 2-methylbutanol and 3-methylbutanol, products of branched chain amino acids, were produced in similar amounts in growing and non-growing cells. Overall, the fact that several flavours were produced at distinctly different levels for growing and non-growing cells establishes the relevance of this developed assay. To show possible interaction between the individual amino acid conversion routes, non-growing cells of *L. lactis* 1157 were incubated with either single amino acids or a mixture of 18 amino acids. Clear differences were observed in the absolute amounts of individual flavour components and ratio between the various flavour components when single substrates or substrate mixtures were used. Some volatile metabolites were only produced upon incubation with single amino acids, while others were only produced in the complete amino acid mix (Table 3). Some of the observed volatile compounds appeared to be uniquely produced from a specific amino acid, such as 3-methylpentanal, 3-methylbutylvalerate, 2,6-nonadienal and 5-methyl-2-isopropyl-2-hexenal from leucine, benzaldehyde and phenylacetaldehyde from phenylalanine and methanethiol, dimethylsulfide and dimethyldisulfide from methionine. However, compounds such as 3-methylbutanal and 2-methylbutanal which are usually produced from branched-chain amino acids [41], were also formed upon incubation with aromatic amino acids and with methionine although the concentration in absence of leucine is 100 – 300-fold lower. Interestingly, some of the compounds observed in single amino acid incubations – methanethiol, dimethylsulfide, dimethyldisulfide, 3-methylpentanal – were not found in the incubations with the complete amino acid mix. For instance, sulphur containing products such as dimethyldisulfide, dimethyltrisulfide and methanethiol were only found when methionine was offered as the single amino acid substrate. This phenomenon was also observed when phenylalanine was the single amino acid substrate, but also uniquely benzaldehyde production was observed. Apparently, the different amino acid converting enzymes, and most probably the amino-transferases, seem to compete for their individual amino acid substrates in this model system. Such substrate interactions and competitions will probably also occur during complicated food fermentations and can be elucidated using an experimental set-up as described here. If enzyme or subtrate competition occurs during cheese ripening it may have a huge impact on developing the flavour profile during ripening. We also confirmed that flavour formation is highly strain- and growth phase-dependent. In previous studies using growing cells of *L. lactis* SK11 and MG1363, it was shown that these strains were not capable of producing 3-methylbutanal in M17-medium [43]. However, in the current study we found that these strains actually are able to produce this relevant flavour compound under non-growing conditions. Two of the *L. lactis* strains in this study (MG1363 and IL1403) produced significantly lower amounts of volatiles compared to the other three strains tested. Both these low-flavour-producing strains are well-studied, plasmid-free, model strains [44,45]. While other explanations are possible, this suggests that some flavour-generating activities are encoded on lactococcal plasmids, a phenomenon that is currently being investigated. Overall, this data clearly confirm that all these flavour compounds are actually produced through the conversion of amino acids and not from any other substrate since these were not made available in the assays. Ayad [46] reported production of the alcohols, ethanol, 2-methyl propanol in cheese paste or in a milk culture by strain *L.lactis* 1157 which is consistent with the results, with the addition that we now show that 2-methylpropanol is exclusively produced under non-growing conditions (Table 2). Ayad also reported the production of the aldehydes 2-methylpropanal, 2- methylbutanal, 3-methylbutanal and benzaldehyde. In addition to that finding we have found that 2-methylpropanal is produced at a two-fold higher level and benzaldehyde at a five-fold higher level under non-growing conditions, while 3-methylbutanal and 2-methylbutanal are produced in similar quantities under growing and non-growing conditions. Furthermore, the results show exclusive acetone, diacetyl, butanone and DMDS production under growing conditions support the high levels observed in fermented milk cultures and lower levels in cheese paste incubations reported by Ayad. The observed differences between the cheese paste cultivation and buffer incubations could be explained by the absolute absence of any carbon source in our case and the presence of citrate as additional substrate in the case of the cheese paste model. Using a microcheese model where different strains of *L. lactis* were added as adjunct cultures in Gouda cheese production, Bachmann found increased production of diacetyl using strains IL1403 (78%) and SK11 (25%), but not using strain 1157, and increased production of 3-methylbutanal using strains 1157 and SK11 which is consistent with our results. These comparisons with actual, experimental, dairy products demonstrate that this rapid method for studying flavour formation by non-growing lactic acid bacteria provides relevant flavour information for actual production of cheese and other fermented dairy products. Furthermore it enables the study of single amino acid conversions and can be used as tool to compare production of flavour components under growing and non-growing conditions.

**Table 2:** **Flavour production at different growth stages**

	**1157**		
	**Non-growing**	**Growing**	**Ratio (n-gr/gr)**
2-butanone	n.d.^a^	7.2 × 10^7^	-^b^
Acetic acid	n.d.	4.3 × 10^6^	-
Acetone	n.d.	8.7 × 10^7^	-
Diacetyl	n.d.	6.4 × 10^7^	-
DMDS	n.d.	3.0 × 10^7^	-
2-methylpropanol	8.7 × 10^7^	n.d.	+
n-butyl isovalerate	2.6 × 10^6^	n.d	+
3-methylbutyl-2-methylbutyrate	1.1 × 10^7^	n.d	+
Methylbenzoate	9.1 × 10^5^	n.d	+
2-isopropyl-5-methyl-2-hexanal	2.9 × 10^7^	n.d	+
4-methyl-2-phenyl-2-hexanal	1.1 × 10^7^	n.d	+
2-methylpropanal	8.9 × 10^8^	5.0 × 10^8^	2
3-methylbutanal	9.5 × 10^8^	8.7 × 10^8^	1
2-methylbutanal	8.8 × 10^8^	8.7 × 10^8^	1
3-methylbutanol	1.2 × 10^8^	2.7 × 10^8^	0.5
2-methylbutanol	1.7 × 10^8^	9.4 × 10^7^	2
Benzaldehyde	1.8 × 10^7^	4.0 × 10^6^	5
Phenylacetaldehyde	2.3 × 10^8^	2.6 × 10^7^	9
5-methyl-2-phenyl-2-hexenal	1.7 × 10^8^	2.5 × 10^6^	70

Volatile compound production (GCMS-peak area^a,b^) of incubations of *L. lactis* 1157 during growth in CDM and under non-growing conditions in a buffer system containing 100 mM sodium phosphate, pH 6.0, 2 mM NADH, 10 mM α-ketoglutarate, 0.1 mM pyridoxal phosphate and a mixture of 18 amino acids.

^a^n.d, not detected.

^b^- and +, not produced or only produced by non-growing cells in comparison to growing cells respectively.

**Table 3:** **Amino acid dependent flavour production**

	**Methionine**	**Leucine**	**Phenylalanine**	**Tyrosine**	**aa-mix**	**Reference**
2-methylpropanal	1.0 × 10^8^	9.5 × 10^7^	1.2 × 10^7^	2.2 × 10^7^	5.7 × 10^9^	n.d.^a^
2-methylpropanol	n.d	n.d.	n.d.	n.d.	1.2 × 10^8^	n.d
3-methylbutanal	4.3 × 10^8^	4.0 × 10^9^	1.3 × 10^8^	1.3 × 10^8^	2.2 × 10^9^	n.d.
2-methylbutanal	2.5 × 10^8^	5.2 × 10^8^	4.6 × 10^7^	6.0 × 10^7^	1.5 × 10^9^	n.d.
DMDS	8.3 × 10^7^	n.d.	n.d.	n.d.	n.d.	n.d.
3-methylpentanal	n.d.	3.8 × 10^7^	n.d.	n.d.	n.d.	n.d.
Methional	5.2 × 10^8^	n.d.	n.d.	n.d.	n.d.	n.d.
2,6-nonadienal	n.d.	4.1 × 10^8^	n.d.	n.d.	5.7 × 10^7^	n.d.
DMTS	1.1 × 10^7^	n.d.	n.d.	n.d.	n.d.	n.d.
Benzaldehyde	n.d.	n.d.	3.2 × 10^8^	n.d.	n.d.	n.d.
Phenylacetaldehyde	n.d.	n.d.	3.1 × 10^9^	n.d.	5.8 × 10^8^	n.d.
3-methylbutylvalerate	n.d.	9.5 × 10^7^	n.d.	n.d.	1.4 × 10^7^	n.d.
5-methyl-2-isopropyl-2-hexenal	n.d.	2.0 × 10^9^	n.d.	n.d.	3.8 × 10^7^	n.d.

Volatile compound production (GCMS-peak area^a^) of incubations of *L. lactis* 1157 in a buffer system containing 100 mM sodium phosphate, pH 6.0, 2 mM NADH, 10 mM α-ketoglutarate, 0.1 mM pyridoxal phosphate, 10 mM methionine, 10 mM tyrosine, 10 mM phenylalanine, 10 mM leucine or 18 amino acids where 13 volatiles are presented (AA-mix).

^a^n.d, not detected.

The reference is the complete incubation mixture without amino acids.

## Conclusions

The majority of flavour production in fermented foods, including cheese, occurs during the extended non-growing phase of lactic acid bacteria. We have chosen to use and further develop an existing experimental method to study the role of *Lactococcus lactis* in cheese flavour production. The method uses active cells, harvested in the stationary growth phase, and resuspended at high concentrations in physiological buffer, at relevant pH-values with the addition of several co-factors known to be involved in flavour production such as NADH, α-ketoglutarate and pyridoxal-phosphate. We use the method to demonstrate that; 1. There are major differences in amino acid conversion by different (non-growing) *L. lactis* strain resulting in large variations in flavour profiles, 2. The conditions of the *L. lactis* cells (growing versus non-growing) has a major impact on their flavour production, 3. The conversion of amino acid mixtures is not just a simple addition of the conversion reaction of each single amino acid 4. The use of concentrated suspensions of non-growing *L. lactis* cells is a suitable method to study flavour production during ripening of semi-hard cheeses, and 5. The method can be used for rapid evaluation of flavour formation since it allows the use of concentrated cell suspensions.

## Methods

### Bacterial strains and concentrated strain stocks

*Lactococcus lactis* strains MG1363 [45], SK11 [47] and KF147 [48], as well as *L. lactis* strains IL1403 [44] and 1157 [46] were routinely cultivated in M17-medium containing 0.5 % glucose (MG1363 and IL1403) or 0.5% lactose (KF147, SK11 and 1157). After overnight incubation (30°C), stationary phase cells were harvested by centrifugation at 6000 × g at 4°C. Cells were washed twice with 100 mM sodium phosphate buffer (pH 6.0) and resuspended in the same buffer with 15% glycerol at an optical density of 400 at 600 nm (OD_600_). Aliquots of these cell suspensions were quickly frozen in liquid nitrogen and stored at −40°C.

When experiments were performed with growing cells, *L. lactis* strain 1157 was incubated in Chemical Defined Medium (CDM) [49] with lactose as carbon and energy source. The cultures were harvested, as described earlier, by centrifugation just before entering the stationary phase.

### Buffer incubation

Volatile production by non-growing cells was studied in 100 mM sodium phosphate buffer (pH6.0) with various additions. α-ketoglutarate (10 mM), NADH (0.2 mM), pyridoxal-5-phosphate (0.1 mM) and 18 amino acids; alanine, arginine, aspartic acid, cysteïne, glutamic acid, histidine, isoleucine, leucine, lysine, phenylalanine, proline, serine, threonine, tryptophan, valine, glycine, tyrosine and methionine (10 mM) were added to the cell suspensions when incubated in this buffer system. After thawing, concentrated cells were added to a final OD_600_ of 40 in the incubation mix. GC vials of 10 ml were filled with 3 ml incubation mixtures and closed with caps to prevent evaporation of volatile compounds. To correct for chemical formation of volatiles, incubation mixtures without cells were performed as controls. Incubations with growing cells were performed in biological triplicates, whereas buffer incubations with single amino acids were performed as a single incubation. This was the consequence of the limited number of samples which could be measured in one GCMS-run and we preferred more variation in incubation conditions and strains used over duplicate or triplicate analysis. From previous fermentation work in our laboratory involving flavour-generation [50] we built sufficient confidence in the high reproducibility in product formation of duplicate and triplicate samples to allow such as choice. After incubation for 24 h at 30°C, the incubation was terminated by flash-freezing of the vials in liquid nitrogen. The vials were stored at −40°C prior to flavour analysis.

### GC-MS analysis

Volatile compounds were measured in the headspace of the incubated samples with the use of Headspace Solid Phase Dynamic Extraction (HS-SPDE) method coupled to a gas chromatograph (trace GC ultra; Thermo Scientific) and mass spectrometer (Thermo Scientific) using a source temperature of 280°C and a transferline temperature of 250°C. After equilibration for 10 minutes at 60°C, the volatile compounds were concentrated onto the phase of the needle by introducing the sample repeatedly through the needle (stationary phase PDMS/AC). The compounds were then desorbed from the syringe needle into the injector (250°C) followed by cryo fixation at −110°C onto the beginning of the analytical column. The cold trap was rapidly heated to 250°C and the compounds were separated on a VF-1 ms column. (Varian, CP8930: 60 m × 0.32 mm, DF = 1,0 μm). The column temperature was initially 40°C for 2 minutes, then gradually raised to 250°C with 10°C/min and subsequently held at that temperature for 5 minutes. The mass spectrometer was operated in a full scanning mode (m/z 25–250) using electron impact ionization (70 eV). The carrier gas was helium, with a constant flow of 1,5 ml/min. Compound structures were assigned by retention time index and the MS spectrum interpretation, quantification and comparison of the spectra were performed using the bibliographic NIST/EPA/NIH Mass Spectral Library (NIST, USA) and Wiley Mass Spectral Library (John Wiley and Sons, USA).
